# Evaluating automated evaluation systems for spoken English proficiency: An exploratory comparative study with human raters

**DOI:** 10.1371/journal.pone.0320811

**Published:** 2025-03-28

**Authors:** Tianhui Chen, Sanjun Sun

**Affiliations:** 1 City Culture and Communication College, Suzhou City University, Suzhou, Jiangsu Province, China; 2 School of English and International Studies, Beijing Foreign Studies University, Beijing, China; Faculty of Electrical Engineering, Computer Science and Information Technology Osijek, CROATIA

## Abstract

Automated evaluation systems (AESs) for spoken language assessment are increasingly adopted in global educational settings, yet their validity in non-Western contexts remains underexplored. This study addresses this gap by examining three widely used Chinese-developed AES tools in their assessment of spoken English proficiency among 30 Chinese undergraduates. The study employed an IELTS-adapted speaking test, assessed simultaneously by AESs and human raters, with scoring alignment analyzed through intra-class correlation coefficients, Pearson correlations, and linear regression. Results revealed that two systems demonstrated strong agreement with human ratings, while the third exhibited systematic score inflation, likely due to algorithmic discrepancies and limited consideration of nuanced language features. Our findings suggest the potential of AESs as valuable complements to traditional language assessment methods, while highlighting the necessity for calibration and validation procedures. This research has significant implications for integrating AESs in educational contexts, particularly in English as a Foreign Language (EFL) settings, where they can enhance efficiency and standardization.

## Introduction

The assessment of spoken language proficiency plays a crucial role in language education and certification, with significant implications for academic and professional opportunities globally [1]. Traditional human judgment, while valuable, can be time-consuming, inconsistent, and resource-intensive [2]. Recent technological advancements have led to the development of automated evaluation systems (AESs) for spoken language evaluation, which employ speech recognition, natural language processing, and machine learning [3].

The integration of AESs into high-stakes language assessment raises questions about their reliability and validity when compared to human rater standards [4]. While AESs offer potential benefits, their performance across diverse contexts requires ongoing investigation. Therefore, it is crucial to ensure the effectiveness and applicability of AESs through continuous evaluation, particularly among learners from non-English-speaking backgrounds [5].

In China, the demand for efficient and accurate spoken English assessment has intensified [6], driving the development of AESs tailored to Chinese learners. However, the efficacy of these systems—defined as their accuracy, reliability, and applicability in assessing spoken English skills—specifically among learners from diverse academic disciplines within Chinese universities, remains understudied. This study, therefore, aims to evaluate the validity of AESs in different contexts to better understand their effectiveness and limitations.

To comprehensively evaluate the reliability and accuracy of AESs in comparison to human ratings across different disciplines, this study examines three representative locally developed AESs from China: AI Speaking Master, TalkAI Language Practice, and SmartSpeech AI Assessment. These systems were selected for their widespread use and high user satisfaction in the Chinese market, offering valuable insights into the performance of commercially successful AESs in assessing spoken English proficiency among non-native English-speaking undergraduates across various disciplines in Chinese universities.

Our research seeks to answer:

(1) How does the overall performance of these AESs compare to human raters across different academic disciplines?
(2) What are the strengths and limitations of each system based on alignment with human scores?
(3) What are the implications for potential implementation in Chinese higher education and similar environments?

By addressing these questions, this study contributes to the growing body of research on automated scoring technologies, with implications that extend beyond the Chinese context. The findings aim to inform global efforts in integrating AESs into language assessment practices, providing insights into their scalability, fairness, and alignment with human evaluation standards.

## Literature review

This review presents four key areas relevant to the evaluation of AESs for spoken English proficiency in the Chinese context: theoretical foundations of language assessment, challenges in assessing spoken English proficiency, the development and implementation of AESs, and comparative studies between human raters and automated systems.

### Theoretical foundations of language assessment

Language assessment has evolved significantly, particularly with the integration of technology-assisted methods. The theoretical underpinnings are largely informed by Bachman and Palmer’s [7] model of language ability, which conceptualizes language proficiency as a complex interplay of language knowledge (organizational and pragmatic) and strategic competence. Bachman and Palmer’s framework also emphasizes the multi-faceted features of language capability, including grammar and vocabulary knowledge and the ability to use this knowledge in communicative contexts.

Multiple dimensions must be evaluated to apply this model in spoken language evaluations, such as fluency, vocabulary usage, accuracy, coherence, pronunciation, and grammatical range [8]. Recent research by Bamdev et al. [9] highlights the importance of these features in automated scoring, suggesting that more focused methods may yield better evaluation results. Integrating these insights into Bachman and Palmer’s framework [7] can provide a solid foundation for developing efficient evaluation tools. This multifaceted approach reflects the complexity of spoken language production and comprehension, involving both linguistic knowledge and its application in real-time communication.

Building on Bachman and Palmer’s work, Fulcher [10] emphasized considering the sociocultural context in which language is used, arguing that assessment should reflect authentic communication tasks encountered in real-world situations. This aligns with the growing recognition of the need for assessment practices that consider broader communicative aspects of language use.

Furthermore, Chapelle and Voss [11] proposed a technology-driven evaluation framework, which emphasizes key principles: authenticity, definition, interactiveness, impact, and practicality. Their model offers a comprehensive guideline for developing and implementing AESs, ensuring the alignment of these technologies with the theoretical constructs of language proficiency while addressing practical considerations in diverse educational contexts.

These theoretical foundations provide a critical lens to evaluate the effectiveness of both conventional and automated assessment methods. In this study, “conventional methods” refer to established practices in human rating systems that have been widely used for assessing language proficiency. This highlights the need for assessment tools that capture the nuanced and contextual nature of language use, a challenge that becomes particularly acute in the development of automated systems.

### Spoken language assessment: Challenges and practices

Building upon the theoretical foundations discussed earlier, this section focuses on the practical challenges and limitations specific to spoken language assessment, particularly in the context of evaluating non-native speakers.

### Challenges in assessing spoken English proficiency

Assessing spoken English proficiency presents unique challenges, especially when evaluating non-native speakers in high-stakes contexts. The ephemeral nature of speech necessitates real-time processing and evaluation, requiring assessors to simultaneously evaluate multiple aspects of language production [8].

Maintaining consistency across different speaking tasks is challenging, as various task types may elicit different aspects of language proficiency, leading to inconsistencies in assessment outcomes [12]. Assessing accent and intelligibility poses additional difficulties, particularly for learners from language backgrounds significantly different from English, requiring careful distinction between accent-related features and actual proficiency issues [13,14].

Evaluating socio-cultural and pragmatic competence adds further complexity, as cultural norms and communication styles can significantly influence language use [15]. Test-taker anxiety can also affect speaking performance, potentially masking true proficiency levels, especially in high-stakes testing environments common in EFL contexts like China [16].

Balancing competing aspects of language production, such as fluency and accuracy, requires careful consideration and well-defined evaluation criteria [17]. These multifaceted challenges underscore the need for robust, well-calibrated assessment tools and methods.

### Limitations of conventional human rating methods

Human rating is the cornerstone of spoken English evaluation and plays a significant role in ensuring the quality and reliability of assessments. It allows for precise judgment and can also account for factors such as context, language fluency, and complexity, which are typically difficult to quantify through automated methods. However, human rating has its limitations. Discrepancies among human raters may result from variations in the interpretation and application of assessment criteria due to individual differences, which can be further intensified when evaluating non-native speakers [18].

The time- and resource-intensive nature of human rating poses challenges, particularly for large-scale assessments common in educational systems like China’s [18]. The inherently time-consuming process of listening to, analyzing, and scoring spoken language samples can hinder the timely feedback crucial for effective language acquisition [19].

Human raters are susceptible to fatigue and inconsistency, potentially compromising assessment validity through phenomena like the “halo effect” [20]. The subjective nature of human rating makes achieving high inter-rater reliability challenging, especially when assessing nuanced aspects of language use [21].

These challenges and limitations have spurred interest in alternative approaches, including the development and implementation of AESs, which will be examined in this study in the context of Chinese undergraduate students’ spoken English proficiency.

### Automated evaluation systems for language assessment

AESs represent a significant advancement in language testing, combining linguistic expertise with technology to provide standardized and potentially more objective evaluations of language proficiency. These systems have evolved from simple pattern-matching algorithms to sophisticated models capable of analyzing complex language features [22].

In spoken language assessment, AESs typically analyze various features, including pronunciation, fluency, lexical diversity, grammatical accuracy, and discourse coherence. Techniques such as forced alignment, phoneme recognition, speech rate, pause patterns, type-token ratio, lexical frequency profiles, part-of-speech tagging, syntactic parsing, error detection algorithms, latent semantic analysis, and coreference resolution are used to assess these features [23,24].

Prominent examples of AESs in high-stakes testing contexts include SpeechRater by Educational Testing Service (TOEFL iBT) and Pearson’s Versant test, which demonstrate high correlations with human ratings for spontaneous speech samples [25,26]. AESs offer increased speed, efficiency, consistency, and rapid, detailed feedback, enhancing both summative and formative assessment practices [27].

However, implementing AESs faces challenges, such as capturing the complexity of human language, ensuring alignment with human rating standards, and addressing diverse linguistic and cultural contexts [28].

China has seen a proliferation of AESs for language learning, reflecting the growing demand for English proficiency and technological innovation. Apps like TalkAI Language Practice (https://ttalkai.com/ [cited 2025 Feb 15]) offer AI-powered conversation practice, personalized feedback, and multi-language support, addressing issues like “mute English” among Chinese learners by encouraging regular speaking practice in a low-pressure environment.

Other AESs used in China include locally developed systems (e.g., Speaking English app by iFLYTEK) and adaptations of international platforms (e.g., Duolingo’s English Test) [29]. These systems are often tailored to the specific needs of Chinese learners, considering factors such as L1 interference and cultural communication patterns.

The integration of AESs into China’s educational technology landscape is part of a broader trend towards “AI+Education,” supported by government initiatives aimed at modernizing education and leveraging technology to address educational challenges, including those in language learning [30].

### Comparative studies: Human raters vs. automated systems

Comparative studies are crucial for assessing the performance of AESs against human raters, establishing validity, reliability, and fairness. Several studies have reported high correlations between AESs and human ratings for various aspects of language proficiency. Zechner et al. [31] found strong correlations (r =  0.57–0.68) between SpeechRater scores and human ratings for spontaneous speech samples in the TOEFL iBT test, while Bernstein et al. [25] reported even higher correlations (r =  0.77–0.92) for the Versant test across multiple languages.

However, the strength of these correlations can vary depending on the specific language features being assessed. Wang et al. [26] found that correlations between automated and human scores differ across aspects such as delivery, language use, and topic development. Kang et al. [32] found that AESs tended to use a narrower range of scores compared to human raters, potentially affecting the discrimination of test-takers at extreme proficiency levels.

AESs have exhibited higher inter-rater reliability compared to human raters for objectively measurable features, while human raters have shown superior performance in evaluating subjective aspects [28]. Cross-linguistic and cross-cultural comparisons have revealed varying degrees of agreement between AESs and human raters across different L1 backgrounds, suggesting potential bias in automated systems [33]. Agreement levels also vary based on test-takers’ proficiency levels [9].

Evaluating pragmatic competence and sociolinguistic appropriateness presents a significant challenge for AESs. Human raters can interpret the appropriateness of language use in context, while AESs may struggle to capture these nuanced aspects of communication [15].

As AESs continue to evolve, future research should consider not only how well AESs align with human ratings but also whether they can provide insights into language use that even human raters might miss. In the context of China, where the demand for English language assessment is high and the use of educational technology is rapidly expanding, comparative studies between AESs and human raters take on particular significance. Future research should also examine how well AESs designed for or adapted to the Chinese market perform in relation to both local and international assessment standards.

## Methodology

The present study employed a comparative design to evaluate the performance of three AESs against human raters in assessing the spoken English proficiency of Chinese undergraduate students.

### Participants

The study involved 30 first-year undergraduate students enrolled in a Spoken English course at Suzhou City University in China. All participants were non-English majors and were recruited through convenience sampling from the course taught by one of the researchers. The sample comprised 60% female and 40% male students, and all participants were native Mandarin speakers, aged 18–20 years (M =  19.1, SD =  0.7).

The participants represented various academic disciplines, including Mechanical Engineering, Electrical Engineering, Computer Science, Health Management, Human Resource Management, and Law. This sampling approach aligned with the study’s exploratory goal of evaluating AES performance across learners from different academic disciplines who had varying exposure to and requirements for English language use.

To protect participant privacy, each student was assigned a unique identifier to ensure personal information was excluded from recordings and subsequent analyses. The study followed the university’s Institutional Review Board (IRB) guidelines, and all participants provided written informed consent after receiving a comprehensive explanation of the research objectives, the voluntary nature of participation, confidentiality measures, and the right to withdraw. Since all participants were adults (aged 18–20), parental or guardian consent was not required. As part of the course requirements, participants received course credit for their participation, which was disclosed during recruitment.

### Instruments

#### Speaking prompts.

Speaking proficiency was assessed using structured oral prompts adapted from the Cambridge IELTS 18 speaking test. These prompts were chosen to reflect the IELTS assessment criteria while introducing culturally relevant topics and appropriate cognitive challenges for Chinese learners.

Before the main study, researchers conducted a pilot test with three students whose profiles matched the broader study sample. The pilot test served two purposes: (1) to validate the speaking prompts’ clarity, cultural relevance, and difficulty level, and (2) to refine the assessment procedures, particularly the scheduling and timing of speaking tasks. Based on the pilot participants’ feedback and performance, necessary adjustments were made to the prompts and procedures. Data from these pilot participants were excluded from the final analysis.

Moreover, two experienced EFL educators reviewed the prompts, assessing their relevance and alignment with the research aims. Their expert validation confirmed that the prompts were appropriate and effective in evaluating the language proficiency of Chinese EFL learners.

To avoid biases, the prompts were randomly assigned to participants, ensuring that no external factors influenced their performance, a critical measure for maintaining the integrity of the evaluation process.

The structured assessments were divided into two parts: Part I consisted of introductory questions eliciting spontaneous speech on everyday topics such as online shopping habits, recent purchases, and the impact of e-commerce on urban life in China. Part II required participants to deliver a 1–2 minute monologue about a tourist destination after 1 minute of preparation, assessing their ability to organize and deliver a coherent, complex discourse without interactive communication.

#### Automated evaluation systems.

Three popular Chinese-developed AESs were employed in this study: AI Speaking Master, TalkAI Language Practice, and SmartSpeech AI Assessment. These mobile-based systems were selected for their popularity and high user ratings in the Chinese market, offering insights into the performance of commercially successful AESs.

- AI Speaking Master (version 2.1.5): An Android app offering features such as anytime practice, multi-national accents, and mock exams. (https://vchat.shuwenkeji.cn/ [cited 2025 Feb 15])
- TalkAI Language Practice (version 2.5.8): Available for iOS and Android, supporting over 60 languages and providing AI tutoring and scenario-based exercises. It has received an impressive rating of 4.9 out of 5 from 29,000 reviews on the Apple App Store. (https://ttalkai.com/ [cited 2025 Feb 15])
- SmartSpeech AI Assessment: A WeChat mini-program developed in collaboration with a university, offering comprehensive scoring across multiple dimensions including accuracy, fluency, completeness, stress, and speech rate. (https://smart-speech.com/ [cited 2025 Feb 15])

Each system analyzed and scored the participants’ recorded responses using proprietary algorithms and scoring criteria. While specific algorithmic details are not publicly available, these systems generally employ speech recognition and natural language processing techniques to evaluate spoken language proficiency.

### Procedure

For the main study, the oral assessment was conducted individually for each participant over seven days. Each participant was scheduled for a 30-minute session, beginning with a briefing to introduce the specific procedures and reduce participants’ anxiety, followed by the oral evaluation, and concluding with a debriefing. This structure aimed to provide a consistent experience for all participants, including the same room designation, recording facility, and uniform evaluation criteria to ensure the comparability of the assessment process.

Evaluations were conducted at the same time of day to control for potential performance variations related to time. This approach ensured fairness and consistency across all sessions and minimized external factors that could affect participants’ performance. All recordings were anonymized using third-party software, ensuring effective anonymization and preventing any bias related to participant identity.

To encourage spontaneous responses and reduce the potential impact of prior exposure to the specific test questions, participants were instructed not to share the prompts. This instruction was reinforced through a confidentiality agreement signed by all participants, along with reminders before and after the assessment.

### Data collection and analysis

#### Human rating.

Three experienced English language teachers (two Chinese and one native English speaker), qualified in language assessment, independently rated the participants’ oral performances. Before scoring, the raters participated in calibration sessions to ensure standardized scoring. The scoring used a 100-point scale aligned with IELTS band descriptors for fluency and coherence, lexical resource, grammatical range and accuracy, and pronunciation. This alignment allowed for detailed scoring while maintaining consistency with IELTS assessment criteria. Inter-rater reliability among the human raters was calculated using intra-class correlation coefficients (ICC) to assess scoring consistency. We employed a two-way random-effects model (ICC2) for both single measures and average measures. This model is appropriate when all participants are rated by the same raters, who are considered a random sample from a larger population of similar raters.

#### Automated scoring.

The same recordings were processed by each AES, generating scores for each participant. Consistent recording conditions ensured standardized input across all scoring methods.

#### Comparative analysis.

The averaged human scores served as the benchmark for evaluating the accuracy and reliability of the automated systems. A comparative analysis examined the agreement between automated and human scores using ICCs, Pearson correlation coefficients, and linear regression analyses. These statistical methods were chosen based on Bachman and Palmer’s [7] emphasis on multidimensional language proficiency, allowing researchers to assess how well the AESs align with human raters across various proficiency dimensions.

Before conducting our analyses, we performed a comprehensive assessment of statistical assumptions. Normality was tested using the Shapiro-Wilk test for each rater. We assessed homogeneity of variance with Levene’s test and examined linearity through scatterplot inspections. For regression analyses, we checked for multicollinearity, identified influential outliers using Cook’s distance, and assessed homoscedasticity and the normality of residuals. These steps guided our selection of appropriate statistical tests, using parametric methods when assumptions were met and non-parametric alternatives when violated.

The system demonstrating the closest alignment with human ratings was considered the most reliable, reflecting Chapelle and Voss’s [11] principle of construct definition and authenticity in automated assessments.

## Results & discussion

This section presents our findings on the performance of three AESs—AI Speaking Master, TalkAI Language Practice, and SmartSpeech AI Assessment—in assessing the spoken English proficiency of Chinese undergraduate students. We compare these systems against human rater benchmarks using descriptive statistics, reliability measures, and correlation analyses. Our analysis addresses the key research objectives: evaluating AES reliability and accuracy, identifying discrepancies with human scoring, and examining implications for Chinese EFL assessment practices. We begin by establishing human rater reliability before analyzing each AES’s performance both individually and comparatively.

### Human rater reliability

Establishing a reliable benchmark for evaluating AESs necessitated a thorough examination of consistency among the three human raters. This analysis was particularly crucial given the linguistic and cultural nuances involved in assessing the English proficiency of Chinese learners.

Descriptive statistics revealed a high degree of agreement among raters, with mean scores clustering tightly between 86.30 and 86.57 and standard deviations ranging from 2.90 to 4.15 (Table 1). This narrow variability in scores suggests strong consistency in the human rating process, despite potential challenges in interpreting Chinese learners’ English pronunciation and discourse patterns.

**Table 1:** Descriptive Statistics for Scores by Human Raters.

Rater	Mean Score	SD
Human Rater 1	86.57	2.90
Human Rater 2	86.37	2.92
Human Rater 3	86.30	4.15

To quantify inter-rater reliability, we employed ICC, a robust measure for assessing agreement among multiple raters. The ICC for single measures (ICC2,1) was 0.713 (95% CI: 0.539-0.835), indicating moderate reliability, while the ICC for average measures (ICC2,k) was 0.882 (95% CI: 0.778-0.938), suggesting good reliability. These results demonstrate a high level of agreement among the human raters, providing a solid foundation for evaluating the performance of the AESs.

Further examination revealed varying degrees of agreement among the human raters. The strongest correlation was between Human Rater 1 and Human Rater 2 (r =  0.901, 95% CI [0.800, 0.952], p < .001), while the weakest was between Human Rater 1 and Human Rater 3 (r =  0.583, 95% CI [0.281, 0.779], p < .001). This variability underscores the complexity of spoken language assessment and highlights the potential value of AESs in providing consistent evaluations.

The strong inter-rater reliability achieved is particularly noteworthy in the context of assessing Chinese learners of English. It suggests that the raters successfully navigated potential pitfalls such as L1 interference in pronunciation, culturally influenced discourse organization, and China-specific vocabulary usage. This consistency is crucial for several reasons:

- Reliability of Benchmark: It ensures that the benchmark used to assess the automated systems is reliable and trustworthy, essential for drawing valid conclusions about the accuracy and effectiveness of these systems in the Chinese context.
- Effectiveness of Rating Criteria: The high consistency suggests that the rating criteria and training procedures were effective in promoting uniformity among raters, even when dealing with the unique characteristics of Chinese learners’ English.
- Bridging Cultural Gaps: The strong agreement among raters, including both native Chinese and native English speakers, indicates a successful bridging of potential cultural gaps in assessment perspectives.
- Fairness and Objectivity: This level of consistency is vital for ensuring fairness and objectivity in language assessment, particularly important in high-stakes testing scenarios common in the Chinese educational system.

Overall, this high inter-rater reliability sets a strong foundation for the subsequent analysis of AESs. It provides confidence that any discrepancies observed between human and automated scores are likely due to genuine differences in assessment approach rather than inconsistencies in human rating. In the context of Chinese learners of English, where unique linguistic features and cultural influences can impact language production, this level of human rater consistency is particularly valuable, enhancing the validity of the subsequent AES evaluation.

### Automated evaluation system performance

#### Overall comparison with human raters.

To evaluate the performance of the three AESs in relation to human raters, we conducted a comparative analysis of their scoring patterns. Table 2 presents the descriptive statistics for scores assigned by each AES alongside those of the human raters.

**Table 2:** Descriptive Statistics for Human Raters and AESs.

Rater	Mean Score	SD
Human Raters (Average)	86.41	3.32
AI Speaking Master	86.63	2.97
TalkAI Language Practice	86.17	3.37
SmartSpeech AI Assessment	92.00	2.85

AI Speaking Master and TalkAI Language Practice demonstrated mean scores and standard deviations remarkably similar to those of the human raters. Paired t-tests revealed no significant differences between these systems and the human average scores (p >  0.05 for both), with negligible effect sizes (Cohen’s d =  0.137 and -0.148, respectively). This alignment suggests these systems have effectively calibrated their algorithms to match human rating patterns for Chinese learners of English.

In contrast, SmartSpeech AI Assessment displayed a notably higher mean score (92.00) and a slightly lower standard deviation (2.85), indicating a systematic deviation from both human raters and other automated systems. A paired t-test confirmed a significant difference between SmartSpeech AI Assessment and the human average scores (t(29) =  20.200, p < .001), with a large effect size (Cohen’s d =  3.688) suggesting a substantial practical difference between SmartSpeech’s scores and human ratings.

**Fig 1:**
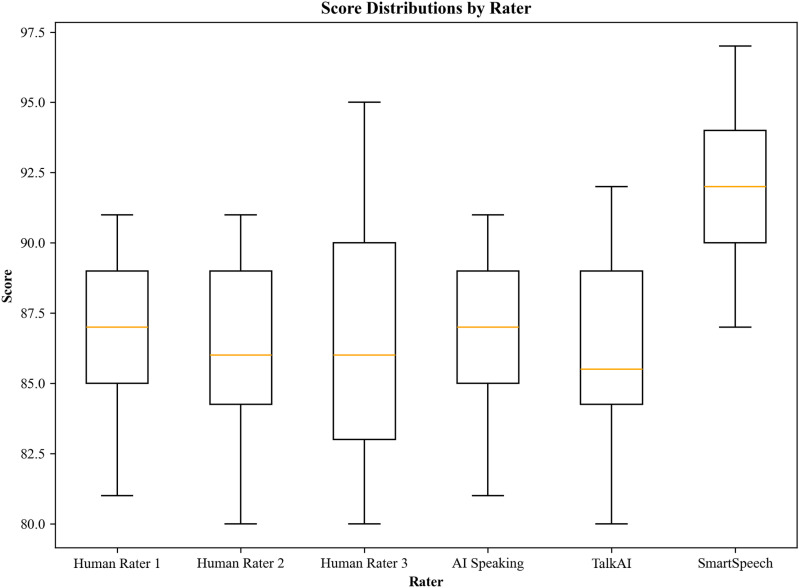
Score Distributions by Rater.

Shapiro-Wilk tests indicated that scores from all human raters and the three systems were normally distributed (p >  0.05). Fig 1 presents boxplots of these score distributions.

The boxplots reveal that AI Speaking Master and TalkAI Language Practice exhibit distributions closely mirroring those of human raters, with comparable medians and interquartile ranges. This similarity suggests that these systems have successfully internalized the nuances of evaluating Chinese learners’ English proficiency. Conversely, SmartSpeech AI Assessment displays a higher median score and a more compressed distribution, indicating a consistent tendency to award higher scores than human raters.

These findings highlight the varying degrees of alignment between AESs and human raters in assessing Chinese EFL learners. While AI Speaking Master and TalkAI Language Practice demonstrate promising congruence with human evaluation standards, SmartSpeech AI Assessment’s consistent score inflation presents a notable deviation. This discrepancy underscores the importance of thorough validation and potential recalibration of automated systems to ensure alignment with established assessment standards.

#### Individual system analysis.

To evaluate each AES comprehensively, we analyzed their agreement with human raters, correlations, and regression results. All three systems demonstrated strong positive correlations with human raters (r =  0.854 to 0.873, p <  0.001) and comparable R-squared values (0.73 to 0.76).

**AI Speaking Master** showed moderate to excellent reliability, with ICC values of 0.737 (95% CI: 0.593-0.845) for single measures and 0.918 (95% CI: 0.854-0.956) for average measures. Its correlation with human raters (r =  0.854, 95% CI [0.714, 0.929], p < .001) suggests strong alignment with human assessment criteria.

**TalkAI Language Practice** demonstrated the highest agreement with human raters, achieving ICC values of 0.750 (95% CI: 0.611-0.854) for single measures and 0.923 (95% CI: 0.862-0.959) for average measures. It showed the strongest correlation with human scores (r =  0.873, 95% CI [0.747, 0.938], p < .001) and the highest R-squared value (0.76), suggesting it most closely mirrors human rating patterns.

**SmartSpeech AI Assessment** presented a unique profile with lower ICC values (0.425 for single measures, 0.747 for average measures) but maintained a strong correlation with human scores (r =  0.869, 95% CI [0.740, 0.936], p < .001). This discrepancy, combined with its systematic score inflation, raises questions about its alignment with human rating standards. The lower ICC, despite a strong correlation, suggests that while SmartSpeech AI Assessment captures the overall trend of the human scores, it deviates significantly in specific instances. This could be due to the system’s potential misinterpretation of features like intonation and hesitation, possibly penalizing natural variations in speech or failing to recognize culturally specific communication styles.

Inter-rater reliability among human raters varied, with correlations ranging from 0.583 to 0.901 (p < .001). The analysis also revealed strong correlations among the three AESs (r =  0.844 to 0.933, p < .001), with AI Speaking Master and TalkAI Language Practice showing the highest inter-system correlation (r =  0.933, 95% CI [0.864, 0.968], p < .001).

These analyses reveal distinct patterns in how each AES aligns with human raters. While AI Speaking Master and TalkAI Language Practice demonstrate strong agreement with human scores, SmartSpeech AI Assessment presents a more complex picture. The consistent performance of these systems suggests promising potential for automated assessment of spoken English proficiency. However, SmartSpeech AI Assessment’s unique scoring pattern highlights the need for careful calibration and interpretation of automated scores, especially in high-stakes contexts.

#### Comparative analysis of AESs.

A comprehensive comparison of the three AESs reveals distinct performance patterns in assessing Chinese EFL learners’ spoken English proficiency. Although the primary aim of this research is to compare the performance of AESs with human raters, it is equally important to assess the comparative performance of different AESs. This comparison has multiple goals: it highlights the diversity of AI assessment methods, identifies which tools perform most consistently in comparison to human evaluation, and outlines the strengths and weaknesses of each tool. Understanding these differences can guide educators and researchers in selecting the most effective language evaluation tool. Table 3 presents the key performance metrics of each system.

**Table 3:** Comparative Performance Metrics of AESs.

Metric	AI Speaking Master	TalkAI Language Practice	SmartSpeech AI Assessment
Mean Score	86.63	86.17	92.00
Standard Deviation	2.97	3.37	2.85
ICC (Single/Average)	0.738/ 0.918 (95% CI: [0.593, 0.845]/ [0.854, 0.956])	0.750/ 0.923 (95% CI: [0.611, 0.854]/ [0.862, 0.959])	0.425/ 0.747 (95% CI: [0.598, 0.847]/ [0.856, 0.957])
Correlation with Human (r)	0.854 [0.714, 0.929]	0.873 [0.747, 0.938]	0.869 [0.740, 0.936]
R-squared	0.73	0.76	0.75

**TalkAI Language Practice** emerged as the most consistent and reliable system, demonstrating the highest ICC values (0.750 for single, 0.923 for average measures) and the strongest correlation with human ratings (r =  0.873, 95% CI [0.747, 0.938]). Its R-squared value (0.76) indicates the highest proportion of variance explained by human ratings, suggesting it has most successfully integrated algorithms mirroring human rater criteria.

**AI Speaking Master** showed slightly lower, but still strong, performance metrics. Its ICC values (0.738 for single, 0.918 for average measures) and correlation with human ratings (r =  0.854, 95% CI [0.714, 0.929]) indicate good to excellent reliability. It demonstrated the lowest standard deviation (2.97), suggesting high scoring consistency valuable for large-scale assessments.

**SmartSpeech AI Assessment** presented a more complex profile, with significant score inflation compared to human raters (mean difference =  5.589, 95% CI: [5.023, 6.155], p < .001). Despite maintaining a strong correlation with human ratings (r =  0.869, 95% CI [0.740, 0.936]), its ICC values were notably lower, particularly for single measures, suggesting substantial deviation from human rating patterns.

Statistical testing revealed no significant differences in correlation coefficients among the three systems (p >  0.05). All correlations between AESs and human raters were statistically significant (p < .001), indicating strong relationships across all systems despite distinct scoring patterns.

These findings highlight the varying approaches and effectiveness of each AES:

- TalkAI Language Practice shows the strongest potential for high-stakes testing scenarios.
- AI Speaking Master offers highly consistent scoring beneficial for large-scale assessments.
- SmartSpeech AI Assessment’s score inflation might be suitable for formative assessments or self-study applications.

The results underscore the importance of careful system selection based on specific assessment needs and contexts, considering factors beyond statistical measures, such as user interface and integration capabilities with existing educational technologies.

### Implications for language assessment

The integration of AESs in language assessment, particularly in the Chinese EFL context, presents significant opportunities and challenges. The strong correlations between AESs and human raters suggest these systems can effectively capture key aspects of language proficiency as outlined by Bachman and Palmer’s [7] model. For instance, AI Speaking Master and TalkAI Language Practice demonstrated high alignment in fluency and grammatical accuracy, supporting the notion that automated systems can reliably assess these multidimensional aspects of language ability. However, variations in correlations underscore the need for careful implementation, ongoing validation, and further research with larger, more diverse samples to differentiate system performances more definitively. This aligns with the findings of Wang et al. [26], who found that correlations between automated and human scores differ across aspects such as delivery, language use, and topic development.

Systematic score inflation observed in SmartSpeech AI Assessment indicates that AI tools still need improvement in capturing more subtle language ability factors, such as pragmatic competence and sociocultural appropriateness. This finding aligns with Chapelle and Voss’s [11] assertion that while technology-driven evaluation shows great potential, it may struggle with tasks requiring a deeper understanding of communicative competence. Furthermore, as Taguchi [15] highlights, evaluating pragmatic competence and sociolinguistic appropriateness presents a significant challenge for AESs, as human raters can interpret the appropriateness of language use in context, while AESs may struggle to capture these nuanced aspects of communication.

Additionally, in the Chinese EFL environment, the high consistency among human raters demonstrates that cultural and social factors are effectively managed in the evaluation process. This is particularly relevant given Fulcher’s [10] emphasis on the sociocultural context in language assessment. The alignment of AESs with human raters in such specific cultural settings highlights their potential effectiveness in similar non-English-speaking environments, provided that they are carefully calibrated to account for sociocultural nuances. As demonstrated in the research by Bernstein et al. [25], the degree of agreement between AESs and human raters can vary across different linguistic and cultural contexts, suggesting potential bias in automated systems.

These findings hold significant theoretical and practical implications for language evaluation. Theoretically, this study contributes to understanding how automated systems can embody key principles of language proficiency theories, despite some limitations. It enhances the integration of technological advancements with established theoretical frameworks to develop comprehensive and effective language assessment tools. Practically, the study provides insights for educational organizations and policymakers globally. It highlights the potential of AESs to improve language evaluation efficiency but cautions against sole reliance on these systems without human oversight, particularly for complex evaluation dimensions closely tied to social and cultural contexts. Future research should focus on expanding the scale and diversity of samples and exploring the ability of AESs to capture pragmatic and sociocultural competencies. Addressing these issues will enable the more effective integration of AESs into language evaluation practices, offering a balanced approach that fully utilizes both technological and human expertise.

## Conclusion

This study offers valuable insights into the performance of three AESs in assessing the spoken English proficiency of Chinese EFL learners. The findings reveal that AI Speaking Master and TalkAI Language Practice demonstrated strong alignment with human raters, while SmartSpeech AI Assessment showed consistent score inflation. These results suggest that AESs can effectively capture key aspects of spoken language proficiency, potentially offering a viable complement to traditional human assessment.

However, the study’s limitations, including the small sample size (N =  30) and narrow range of AESs tested, constrain the statistical power and generalizability of the findings. To address these limitations and advance the field, future research should expand the sample size and diversity, test a wider range of AESs, and investigate performance across different types of speaking tasks and learner proficiency levels.

The integration of AESs in language assessment presents a transformative opportunity for enhancing educational practices, with implications for language education policy, curriculum design, and teacher training. Realizing this potential requires a balanced approach that leverages the strengths of both automated and human evaluation. As the field evolves, continued research, development, and ethical considerations will be essential to ensure that AESs contribute positively to language learning while maintaining assessment validity and fairness.

This balanced approach necessitates a shift towards a more holistic model of language assessment, combining technological efficiency with human insight. As we navigate this technological frontier, maintaining a critical perspective is crucial to ensuring that innovation serves the fundamental goal of enhancing language learning and teaching effectiveness.
